# Prevalence of XMRV Nucleic Acid and Antibody in HIV-1-Infected Men and in Men at Risk for HIV-1 Infection

**DOI:** 10.1155/2011/268214

**Published:** 2011-11-21

**Authors:** J. Spindler, J. Hackett, X. Qiu, A. Wiegand, V. F. Boltz, P. Swanson, J. H. Bream, L. P. Jacobson, X. Li, C. R. Rinaldo, S. M. Wolinsky, J. M. Coffin, M. F. Kearney, J. W. Mellors

**Affiliations:** ^1^HIV Drug Resistance Program, National Cancer Institute, Frederick, MD 21702-1201, USA; ^2^AIDS Research and Retrovirus Discovery, Abbott Laboratories, Abbott Park, IL 60064, USA; ^3^Bloomberg School of Public Health, Johns Hopkins University, Baltimore, MD 21205, USA; ^4^Department of Medicine, University of Pittsburgh, Pittsburgh, PA 15261, USA; ^5^Feinberg School of Medicine, Northwestern University Chicago, IL 60611, USA; ^6^Department of Molecular Biology and Microbiology, Tufts University, Boston, MA 02111, USA

## Abstract

Xenotropic MLV-Related Virus (XMRV) was recently reported to be associated with prostate cancer and chronic fatigue syndrome (CFS). Infection was also reported in 3.7% of healthy individuals. These highly reported frequencies of infection prompted concerns about the possibility of a new, widespread retroviral epidemic. The Multicenter AIDS Cohort Study (MACS) provides an opportunity to assess the prevalence of XMRV infection and its association with HIV-1 infection among men who have sex with men. Reliable detection of XMRV infection requires the application of multiple diagnostic methods, including detection of human antibodies to XMRV and detection of XMRV nucleic acid. We, therefore, tested 332 patient plasma and PBMC samples obtained from recent visits in a subset of patients in the MACS cohort for XMRV antibodies using Abbott prototype ARCHITECT chemiluminescent immunoassays (CMIAs) and for XMRV RNA and proviral DNA using a XMRV single-copy qPCR assay (X-SCA). Although 9 of 332 (2.7%) samples showed low positive reactivity against a single antigen in the CMIA, none of these samples or matched controls were positive for plasma XMRV RNA or PBMC XMRV DNA by X-SCA. Thus, we found no evidence of XMRV infection among men in the MACS regardless of HIV-1 serostatus.

## 1. Introduction

Xenotropic Murine Leukemia Virus-Related Virus (XMRV) is a recently discovered gammaretrovirus reportedly associated with prostate cancer and chronic fatigue syndrome (CFS) [1, 2]. Urisman et al. first identified XMRV in 2006 in a cohort of prostate cancer patients [2], followed by Lombardi et al. who reported XMRV infection in 67% of patients with severe CFS and 3.7% of healthy individuals [1]. These initial reports provided a compelling rationale for further investigations into the prevalence of XMRV infection in human populations. However, controversy arose when subsequent studies failed to detect the virus in similar cohorts [3–7]. It was suggested that inconsistencies in detection of XMRV in patient samples could result from varied incidence of infection in different populations, differing criteria for patient selection, and differing detection methods [8]. It was also proposed that virus levels may be chronically low or episodic in patient plasma or tissues, making virus detection difficult [8]. Adding to the complexity, detection of XMRV by PCR is highly susceptible to false positive results due to amplification of closely related endogenous Murine Leukemia Viruses (MLVs) in the mouse genome and the high prevalence of contaminating mouse genomic DNA in many specimens and reagents [9, 10]. Additionally, studies have suggested that XMRV detection is the result of laboratory contamination from infected cell lines [11–14]. Paprotka et al. proposed that XMRV originated as a laboratory artifact when two endogenous mouse proviruses recombined during passaging of a human prostate cancer tumor in nude mice, an event that is highly unlikely to have occurred more than once. The authors, therefore, concluded that published XMRV sequences obtained from patient samples must have come from contamination of samples by virus or DNA from cell lines infected with this recombinant virus [14]. To investigate the human prevalence of XMRV infection, it is clear that reliable detection requires the application of several diagnostic methods used together, including methods that are not influenced by nucleic acid contamination, to avoid reporting potentially high rates of false positives.

Accordingly, we analyzed recently collected blood samples from participants in the MACS cohort using new tests that detect XMRV antibodies and nucleic acid in the blood stream [15]. The MACS cohort provided the opportunity to assess the association of XMRV with HIV-1 infection and other clinical outcomes and to evaluate its possible mode of transmission. We hypothesized that the prevalence of XMRV infection is higher among men who have acquired HIV-1 infection than among seronegative controls. Previous studies have evaluated samples from HIV-infected cohorts for the presence of XMRV nucleic acid with negative results [7, 16, 17], but none has looked for the presence of antibody to XMRV.

 In the current study, we first screened samples for antibody reactivity to XMRV. This approach eliminated the risk that positive results were due to nucleic acid contamination and mitigated the risk that infection would be missed due to low-level or episodic viremia. To further minimize the risk of reporting false-positive XMRV infection status, we required that antibody and nucleic acid (either viral RNA or DNA) must both be present to report the patient as being XMRV infected. These criteria are supported by studies performed on XMRV-inoculated macaques confirming that both antibody and nucleic acid are readily detectable in longitudinal blood samples collected after XMRV infection (Kearney et al. in press; Del Prete et al. in preparation) [15, 18]. Plasma samples from the MACS cohort were screened for antibody reactivity by CMIAs and confirmed by Western blot. Reactive samples and blinded, matched antibody negative controls from the same cohort were then tested for the presence of XMRV RNA in plasma and proviral DNA in PBMCs. This approach to determine the prevalence of XMRV infection minimizes the risk of reporting false positives that occur from either cross-reactive antibodies in the bloodstream or contaminating nucleic acid in samples or reagents.

## 2. Methods

### 2.1. Clinical Samples

Plasma and PBMC samples were obtained from 332 individuals in the MACS cohort. All samples were collected from clinical visits in 2006–2009. Half of the 332 men were HIV-1 seropositive, of whom 89 were users of antiretroviral therapy (ART) and 77 were ART naïve. HIV-1-seronegative men of similar age, date of study entry, center, and hepatitis status were selected from the same visits as the HIV-1-seropositive men. The median age (interquartiles (25%, 75%)) of both the HIV-1-seropositive and -seronegative men was 50 years (45, 55.5), and the median (IQR) CD4 cell count and HIV-1 RNA levels of the HIV-1 seropositives were 578 (418, 786) cells/mm^3^ and <50 (<50, 12408) copies/mL, respectively. Plasma samples were screened at Abbott Laboratories for the presence of XMRV antibodies by newly developed XMRV CMIAs (ARCHITECT platform) [15]. Samples with positive CMIA results were tested by Western blot [15]. Samples that were positive for XMRV antibodies by CMIA were matched 1 : 3 by age, HIV serostatus, antiretroviral therapy, and pre-ART CD4 cell count for those who had initiated therapy, with CMIA negative samples. All samples were then assayed blinded for the presence of XMRV plasma RNA and proviral DNA in PBMCs using highly sensitive XMRV qPCR assays (X-SCA) (Kearney et al. submitted in press).

### 2.2. XMRV Chemiluminescent Immunoassays (CMIAs) and Western Blot

A detailed procedure has been described previously [15]. Briefly, 100 *μ*L of neat plasma were screened for antibodies to XMRV gp70 (SU) and p15E (TM) proteins using two prototype ARCHITECT chemiluminescent immunoassays (CMIAs; Abbott Diagnostics, Abbott Park, IL). The CMIAs utilize a direct assay format in which *E. coli*-expressed XMRV p15E or mammalian-expressed XMRV gp70 was used as both capture and detection antigens. Assay positive controls were derived from XMRV-infected rhesus macaque plasma at 1 : 1000 (PC1) or 1 : 4000 (PC2). A pool of normal human plasma was used as negative control (NC). Cutoff (CO) values of the ARCHITECT CMIAs were calculated based on the following formulas: CO = 0.45 × (PC2 Mean Relative Light Units (RLU)) for p15E CMIA and CO = 0.078 × (PC1 Mean RLU) for gp70 CMIA. Assay results were reported as the ratio of the sample RLU to the cutoff RLU (S/CO) for each specimen. Specimens with S/CO values <1.00 were considered nonreactive; specimens with S/CO values >1.00 were considered initially reactive. Reactive specimens were further analyzed by ARCHITECT p30 CMIA and by investigational western blot assays. 

 ARCHITECT p30 CMIA also utilizes the direct assay format with *E. coli*-expressed XMRV p30 (capsid protein) to capture and detect anti-p30 antibodies [15]. The same sample volume (100 *μ*L), calibrator, and controls were used for the p30 CMIA. CO of p30 CMIA was calculated based on the formula: CO = 0.27 × (PC1 Mean RLU). 

 Western blot (WB) analysis using purified XMRV viral lysate as well as recombinant gp70 protein was performed as described [15]. Briefly, viral lysate (65 *μ*g/gel) or recombinant gp70 protein (25 *μ*g/gel) was separated by electrophoresis on a 4–12% NuPAGE Bis-Tris 2-dimension gel (Invitrogen, Carlsbad, CA) in the presence of sodium dodecylsulfate (SDS). The protein bands on the gel were electrophoretically transferred to a polyvinylidene difluoride (PVDF) membrane (Invitrogen). After blocking, the PVDF membrane was cut into 2 mm strips. Strips were incubated with human plasma samples diluted 1 : 100 or XMRV-infected macaque plasma [18] diluted 1 : 250 overnight at 2–8°C. After removal of unbound antibodies, strips were incubated with alkaline phosphatase conjugated goat antihuman IgG (Southern Biotech, Birmingham, AL) for 30 minutes at room temperature. The strips were washed, and chromogenic substrate solution was added to visualize the reactive bands.

### 2.3. Nucleic Assay Detection with XMRV Single-Copy Assays (X-SCA)

Similar to the HIV-1 single-copy assay (SCA) [19], real-time PCR and RT-PCR assays for detection of XMRV, called X-SCA (XMRV single-copy assay), were developed to quantify XMRV RNA in plasma and proviral DNA in PBMCs (Kearney et al. in press). X-SCA was designed using amplification primers targeting a conserved* gag *leader region between XMRV and endogenous MLVs allowing detection of both templates. The TaqMan probe was designed to span the signature 15–24 nt deletion (derived from PreXMRV-2 [14] in the XMRV gag leader region compared to other MLV sequences). This probe design results in a lower plateau level of fluorescence from non-XMRV templates than from XMRV templates, thus distinguishing the source of product that is being detected in the assay (Kearney et al. in press). To confirm any positive result, PCR products are run on a 2% agarose gel, allowing distinction between the 86 nt XMRV X-SCA product and the 110 nt non-XMRV product. 

Patient samples from the MACs cohort were tested by X-SCA in triplicate with equal numbers of no template controls (NTC) to monitor the level of false positives due to common mouse genomic DNA contamination. An internal RCAS (avian retrovirus) control was spiked into each plasma sample to quantify nucleic acid recovery from plasma samples as described previously [19]. X-SCA was performed on 0.1-0.2 mL of plasma resulting in a limit of detection of 9–18 RNA copies/mL and on 1 × 10^6^ PBMCs resulting in a limit of detection of 1 DNA copy/1 × 10^6^ cells.

### 2.4. Criteria for Determining XMRV Infection Status

There is a high risk of obtaining false positive results from CMIAs and PCR diagnostic tests [9, 10]. Therefore, we set strict criteria for declaring a sample positive for XMRV. We required that the sample must test positive for both XMRV antibody by CMIAs or Western blot and nucleic acid (either RNA or DNA) by X-SCA. A positive X-SCA test required detection of virus in all triplicate PCR reactions. If discordant results were obtained from triplicate wells, then the result was considered indeterminate.

### 2.5. Data Analysis and Sample Size Calculations

The sample sizes of 200 HIV-1-seropositive and 200 seronegative participants initially were chosen with arcsine approximation to provide 86% power to detect a difference in XMRV prevalence assuming 12% XMRV prevalence in HIV-1 seropositives versus 4% in seronegatives, with *α* = 0.05. The 4% rate among HIV-1 seronegatives corresponded to the 3.7% prevalence observed in published control series [1]. Selection of HIV-1-seropositive men in the current ART era limited the inclusion of ART-naïve men, and the need for unthawed PBMC pellets and EDTA plasma further reduced the sample size to 332. Given the small number of positive results, simple frequencies are used to describe the data.

## 3. Results

### 3.1. XMRV Serology with ARCHITECT CMIAs and Western Blot

Using the direct format ARCHITECT p15E and gp70 CMIAs, XMRV serology was evaluated on 332 recently collected plasma samples from patients in the MACS cohort. Nine samples (5 HIV-1 seropositive, 4 HIV-1 seronegative) were found to be reactive to XMRV proteins, one against the p15E transmembrane protein and 8 against the gp70 envelope protein (Table 1), resulting in a frequency of 0.3% (1/332) positive for p15E and 2.4% (8/332) for gp70. Of note, however, none of the samples were reactive against both p15E and gp70. Subsequent testing of the 9 positive samples with the p30 CMIA showed no detectable anti-p30 antibodies (Table 1).

**Figure 1:**
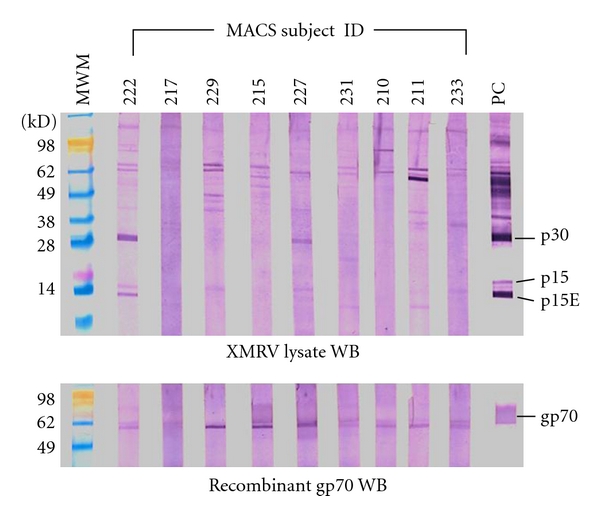
*XMRV Western Blot Analysis.* WB analysis of CMIA reactive MACS subjects using native XMRV viral lysate and recombinant gp70 protein. XMRV-infected macaque plasma was used as positive control (PC). Sample 222 tested positive for p15E by CMIA; all the others were positive for gp70 (Table 1).

 By viral lysate WB, the only p15E CMIA-positive sample 222 was reactive to native p15E protein (Table 1, Figure 1). However, only 1 of the 8 gp70 CMIA-positive samples (215) was reactive in the recombinant gp70 WB. The low confirmation rate could be due to higher sensitivity of the gp70 CMIA or the antibodies detected may primarily recognize conformational epitopes which were sensitive to SDS or thermal denaturation. Although nonreactive in p30 CMIA, two samples, 222 and 227 had an apparent p30 band in the viral lysate WB (Table 1, Figure 1). However, subsequent WB analysis of CMIA-negative normal blood donors showed the presence of p30 band indicative of nonspecificity or cross-reactivity (data not shown). Others have also observed the nonspecific reactivity against XMRV p30 protein in WB [20].

### 3.2. Nucleic Assay Detection with XMRV Single-Copy Assays (X-SCA)

XMRV single-copy assays (X-SCA) were conducted on both plasma and PBMCs from the 9 patients with CMIA-reactive antibodies, and 25 CMIA-negative controls matched for date of MACS entry, HIV serostatus, ART use, time from ART initiation, and pre-ART CD4+ T-cell count (Table 2). None of the samples tested were found to be positive for XMRV nucleic acid by X-SCA (either RNA or DNA) including the 9 samples that were CMIA reactive. Given the sample volumes available for testing, X-SCA had limits of detection for XMRV RNA in plasma of 9.2 copies/mL and 1 XMRV DNA copy in one million PBMCs. Sixty-one copies of RNA were detected in one of triplicates from sample 218, resulting in an indeterminate X-SCA result for this subject. The same sample was CMIA nonreactive, and no XMRV DNA was detected in one million PBMCs. Consequently, the sample did not meet our criteria for a positive XMRV result. A single positive well in an X-SCA run is not above the level of false positives for XMRV real-time PCR assays due to the high frequency of environmental mouse genomic DNA that readily amplifies with the X-SCA primers (Kearney et al. in press).

## 4. Discussion

The XMRV study by Lombardi et al. published in October 2009 suggested a surprisingly high seroprevalence for XMRV, even among healthy control subjects [1]. Therefore, we set out to evaluate the prevalence of XMRV infection in a possible high-risk cohort. We adopted a multiple diagnostic assay approach to determine the XMRV status of patient samples to minimize the possibility of obtaining false positive results by individual molecular or serologic methods. False positive PCR results may occur from incidental amplification of environmental mouse genomic DNA due to the close relationship between XMRV and mouse proviruses [9, 10]. False positive serology results may occur due to nonspecific or cross-reactive human antibodies [15]. In fact, a recent study reported that ~4% of HTLV-I-infected individuals had antibodies that were cross-reactive to XMRV p15E protein [21]. Therefore, we applied stringent criteria for XMRV positivity aimed at limiting the risk of reporting false positive results. Our criteria for an XMRV infection required that (1) all replicates from X-SCA must be positive, (2) antibodies must be detectable by CMIA and/or Western blot, and (3) nucleic acid and antibody must both be positive. Samples resulting in discordant results from X-SCA replicates were reported as indeterminate. Applying these criteria, we did not identify any samples from the MACS cohort that were consistent with XMRV infection. Nine samples had positive serology, but no detectable XMRV nucleic acid. Although one sample was indeterminate for XMRV RNA by X-SCA, it was antibody negative. XMRV DNA was not detected in any sample tested. Because we did not find any patient to be reactive to multiple XMRV antigens and because all nine patients for whom serum reactivity was observed were negative for XMRV nucleic acid, we concluded that follow-up testing by additional methods, such as virus culture, was not necessary. We believe that the combined approach of sensitive antibody screening and sensitive and specific nucleic acid testing excludes XMRV infection in the cohort studied. 

Previous studies have shown that X-SCA is able to detect XMRV RNA and DNA in spiked control samples and in specimens from inoculated macaques with high sensitivity [22] (Kearney et al. in press; Del Prete et al. in preparation). In the samples analyzed here, the sensitivity for detection was limited by the small sample volumes available for testing. Despite this, the limits of detection were still adequate to detect XMRV DNA that has been shown to persist in macaque PBMC samples following XMRV infection (Del Prete et al. in preparation). Consequently, proviral DNA would likely have been detectable in any subject recently infected with XMRV. It has been argued that XMRV nucleic acid could be missed in clinical samples from infected individuals due to low-level or episodic viremia. By testing a large cohort of HIV-infected individuals for immunity to XMRV and not for viral nucleic acid alone, we reduced the risk of reporting false negatives for XMRV infection. 

 Despite earlier reports that evidence of XMRV infection was detected in about 20% of prostate cancer patients [2, 23–25] and 67% of CFS patients [1], we did not find clear evidence for XMRV infection in a cohort of men with HIV-1 infection or at high risk for HIV-1 infection. These results are consistent with prior reports that failed to detect XMRV nucleic acid in HIV-1-infected patients [7, 16, 17, 26]. 

Findings from previous studies reporting higher prevalence for XMRV infection in cohorts typically involved testing by a single diagnostic method. In the current study, if we had based XMRV infection on a single diagnostic method (either PCR or serology), the apparent XMRV prevalence would have been 1.2%; 0.3% (1/332) by PCR and 0.9% (3/332) by serology. Given the potential for false positive results in PCR and serological assays for XMRV, our results suggest that applying multiple diagnostic methods including measuring levels of proviral DNA in blood cells provides a more reliable approach for investigating the prevalence of XMRV infection. We hypothesized that the prevalence of XMRV infection would be higher among men who have acquired HIV-1 infection than among seronegative controls. The negative data from our study clearly refute this hypothesis. Individuals at risk for HIV-1 infection and sexually transmitted infections are not at risk for XMRV infection.

## Figures and Tables

**Table 1:** XMRV antibody reactivity for the MACS Subjects.

MACS ID	CMIA (S/CO)			WB
	p15E TM	gp70 SU	p30* CA	
222	*1.67*	0.08	0.23	p15E+, p30+
217	0.22	*28.48*	0.17	
229	0.18	*8.73*	0.19	
215	0.17	*21.36*	0.21	gp70+
227	0.20	*2.27*	0.18	p30+
231	0.17	*1.56*	0.18	
210	0.18	*1.65*	0.22	
211	0.17	*1.47*	0.20	
233	0.19	*1.80*	0.20	

*Control*				

NC	0.17	0.11	0.23	
PC1	*7.90*	*12.82*	*3.70*	gp70+, p15E+, p30+
PC2	*2.22*	*3.47*	*1.10*	NA

*Samples were tested at 1 : 2.5–5 dilutions due to limited sample volume.

**Table 2:** XMRV nucleic acid and antibody results.

Subject ID	XMRV average RNA copies/mL by X-SCA	XMRV DNA copies per 1e6 cells by X-SCA	ARCHITECT antibody serology	Western blot	XMRV status
201	<10	<1	neg	NT	neg
202	<10	<1	neg	NT	neg
203	<10	<1	neg	NT	neg
204	<9	<1	neg	NT	neg
205	<18	<1	neg	NT	neg
206	<9	<1	neg	NT	neg
207	<9	<1	neg	NT	neg
208	<9	<1	neg	NT	neg
209	<9	<1	neg	NT	neg
210	<9	<1	*gp70+*	*neg*	neg
211	<9	<1	*gp70+*	*neg*	neg
212	<9	<1	neg	NT	neg
213	<9	<1	neg	NT	neg
214	<9	<1	neg	NT	neg
215	<18	<1	*gp70+*	*gp70+*	neg
216	<9	<1	neg	NT	neg
217	<9	<1	*gp70+*	*neg*	neg
218	*61*	<1	neg	NT	neg
219	<9	<1	neg	NT	neg
220	<9	<1	neg	NT	neg
221	<9	<1	neg	NT	neg
222	<9	<1	*p15E+*	*p15E+, p30+*	neg
223	<9	<1	neg	NT	neg
224	<9	<1	neg	NT	neg
225	<9	<1	neg	NT	neg
226	<9	<1	neg	NT	neg
227	<9	<1	*gp70+*	*p30+*	neg
228	<9	<1	neg	NT	neg
229	<9	<1	*gp70+*	*neg*	neg
230	<9	<1	neg	NT	neg
231	<9	<1	*gp70+*	*neg*	neg
232	<9	<1	neg	NT	neg
233	<9	<1	*gp70+*	*neg*	neg
234	<9	<1	neg	NT	neg

*NT indicates that MACS ID was not tested by Western blot assay.
